# Case report – midfoot pain in a collegiate athlete with polycystic ovaries: digging deeper into bones and hormones

**DOI:** 10.17159/2078-516X/2025/v37i1a20221

**Published:** 2025-07-15

**Authors:** JER Henderickx, PL Viviers, JH Kirby

**Affiliations:** 1 Institute of Sport and Exercise Medicine, Department of Exercise, Sport and Lifestyle Medicine, Faculty of Medicine of Health Sciences, Stellenbosch University, South Africa; 2 Campus Health Service, Stellenbosch University, South Africa; 3 Catholic University of Leuven, Department of Physical Medicine and Rehabilitation, Leuven, Belgium

**Keywords:** accessory navicular, polycystic ovaries, bone health

## Abstract

A 22-year-old female ultimate frisbee player, known with polycystic ovary syndrome (PCOS), presented with chronic midfoot pain. Suspecting a bone stress injury, imaging showed bone marrow oedema of the navicular and accessory navicular bones. Conservative management included anti-inflammatories, load reduction, physiotherapy, orthotics and nutritional support. This case discusses the accessory navicular bone and highlights the significance of the interplay between PCOS and bone health in female athletes. Understanding these factors is important for injury prevention and optimal management in female athletes with PCOS.

## Case report

A 22-year-old female ultimate frisbee player presented with gradual onset right foot pain over two months. Walking and running aggravated the midfoot pain, described as a combination of localised constant dull and stabbing pain, so she avoided these activities. On return to campus, walking was unavoidable, and the pain progressed until she was unable to weight-bear for four days before the presentation. Neither physiotherapy nor analgesia and anti-inflammatories resolved the pain. She had resorted to a moon boot and crutches and was concerned about the possibility of a bone stress injury.

In the months preceding her foot pain, anaemia had forced her to reduce her cardiovascular training load, and she had taken up yoga. The anaemia was resolved (haemoglobin 13.9 g/dl), but she had not regained her fitness due to the foot pain. At the time of the consultation, she was taking a multivitamin. One year before presentation, she was diagnosed with polycystic ovary syndrome (PCOS) and reported 16 months of amenorrhea. Combined oral contraceptives had returned her menstrual cycle to normal. Metformin was recently added to her PCOS management to treat her insulin resistance.

On examination, neither swelling nor bruising was observed. The sustentaculum tali and navicular bones were tender to palpation. Neurovascular examination was normal. Foot inversion/eversion and ankle plantarflexion/dorsiflexion were normal, with a neutral medial arch and all tendons intact. Posture, alignment, and movement patterns were normal. The body mass index (BMI) was 26.9 kg/m^2^.

After clinical examination, the differential diagnosis was a navicular stress injury, cuboid syndrome, Lisfranc injury and tibialis posterior tendinopathy, with stress injury being most likely in light of the mechanism of injury and clinical presentation.

**Fig. 1:**
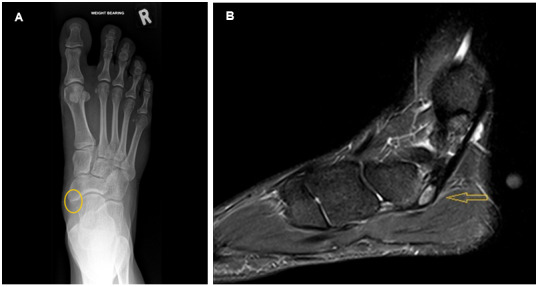
A: Dorsoplantar X-ray view demonstrates the ossicle (yellow circle). B: MRI showing oedematous accessory navicular bone (yellow arrow) and oedema of the medial aspect of the navicular bone.

The Plain film radiographs (Figure 1A) showed an ossicle medial to the navicular bone with overlying soft tissue swelling. Between the bases of the first and second metatarsals 5.4 millimetres were measured, this was flagged by the radiologist, suggestive of a potential ligamentous Lisfranc injury. Magnetic Resonance Imaging (MRI) was advised. As a bone stress injury remained high on the differential, it was requested. In Figure 1B, a T2 fat-saturated proton density sagittal view showed the accessory navicular bone with oedema of the synchondrosis and bone marrow oedema of the ossicle and the medial navicular bone. The tibialis posterior tendon was normal. Surrounding ligaments, including the Lisfranc ligament, and tendons were intact.

A conservative approach with clear patient-reported outcome measures (including pain level, functionality, and return to sport) was implemented following a shared decision-making process. The conservative approach included physiotherapy and analgesia for pain management, a temporary foot orthosis, and strengthening of the intrinsics of the feet. Furthermore, dietary calcium intake was optimised. At present, she has gradually returned to running and is now again participating in ultimate frisbee, six months after the first visit and eight months after injury.

## Discussion

In chronic midfoot pain, the differential diagnosis includes soft tissue overuse injuries (e.g., Lisfranc ligament sprain injury, flexor hallucis longus tendinopathy, tibialis posterior tendinopathy, peroneal tendinopathy), bone stress injuries, osteoarthritis, accessory navicular syndrome, and plantar nerve entrapments (medial, lateral).

In our case, it is a combination of an accessory navicular bone and a bone stress injury, necessitating a deeper look into the risk factors, including relative energy deficiency syndrome. However, our patient was overweight and had PCOS. In this discussion, we will delve into accessory navicular syndromes and whether PCOS can be considered a risk for bone stress injury.

An accessory navicular syndrome is a common additional ossicle (os) of the foot, also known as navicular secundum, os navicular secundarium, os tibial externum, accessory scaphoid, accessory tarsal scaphoid, divided navicular and prehallux.[1] Incidence varies from 4–21% in the general population,[1,2] with evidence suggesting a higher prevalence in females and is more common bilaterally.[2] According to Kalbouneh *et al*.[1] the incidence is estimated to be 21% in patients with chronic midfoot pain. However, the pain was only attributed to the accessory navicular bone in 11% of these patients.[1] Based on the Coughlin classification[2], type I is a small, round ossicle within the tibialis posterior tendon near its insertion and is commonly asymptomatic. Type II is a larger, triangular fragment connected to the navicular tuberosity by a cartilaginous synchondrosis. When attached to the talar process by a less acute angle, it is a type IIA. Type IIB is situated more inferiorly, as it is in this case. Fusion with the navicular bone, representing a cornuate-shaped bone, is called a type III accessory navicular bone. Accessory navicular bone is often detected incidentally on radiographs, but it can cause midfoot pain, weakness and decreased foot function. Symptoms are most common in type II accessory navicular bone owing to altered biomechanics.[1] For instance, the type IIB accessory navicular bone creates abnormal forces due to its fibrocartilaginous connection to the main navicular. These forces can lead to stress on adjacent bones and soft tissues. One can also assume that the function of the tibialis posterior tendon can be disrupted, causing increased stress on the medial longitudinal arch. However, to date, no studies have investigated the link between accessory navicular bones and bone stress injuries. Furthermore, accessory navicular bone seems to be associated with pes planus deformity, but whether there is a causal link is still under discussion.[1,2] Only symptomatic accessory navicular bones require intervention, which is mostly conservative.

Bone marrow oedema on MRI results from accumulated interstitial fluid[3] caused by capillary leakage.[4] Trauma by impaction, shear or avulsion is the most common cause of bone bruising and can be acute or chronic due to repetitive loading,[4] as is the case with bone stress injuries.

When suspecting a bone stress injury in an athlete, it is important to identify internal and external risk factors for further management and injury prevention, such as physical activity and stage of training, biomechanical factors, early sport specialization, equipment (shoes and inserts), medication, nutrition, sleep disorders and genetic factors.[3] Since bone stress injuries occur more often in female athletes, sex-specific risk factors have been investigated, like hormonal disturbances,[3] as is the case with PCOS.

PCOS was formerly thought to protect against bone mineral density (BMD) loss due to hyperandrogenism and a higher BMI. Androgens can be converted to oestrogens and, in doing so, positively impact bone health. However, studies examining the link between BMD and PCOS have shown inconsistent results.[5] New research suggests that chronic inflammation, insulin resistance, metabolic abnormalities, and vitamin D deficiency could play a role in decreased BMD[5,6] in PCOS. Both seem related through complex biological mechanisms and pathways influenced by genetic and epigenetic factors.[5]

Inadequate insulin signalling can influence bone remodelling by reducing bone formation and increasing bone resorption.[5] Mechanisms being investigated are decreased osteoprotegerin (OPG) expression, an inhibitor of a member of the tumour necrosis factor family (RANKL) involved in bone resorption, and insulin receptor expression in osteoclasts impacting their recruitment.[5] However, the precise mechanism is still not fully understood, and the clinical significance is unclear, which makes further research on the link between PCOS, osteoporosis and insulin necessary.

In addition, chronic inflammation and oxidative stress in PCOS may compromise bone strength, even in the presence of elevated androgen levels and increased body weight.[5] Oxidative stress, exacerbated by DNA damage, mitochondrial dysfunction, hormonal imbalances and insulin resistance, deteriorates bone quality.[5]

The insulin signalling and inflammatory pathways both seem to play a significant role, so understanding these processes is critical for further managing bone health in PCOS patients. Considering a potential link between PCOS and bone stress injuries, one could assume that women with PCOS have a higher risk for bone stress injuries due to the preceding factors. However, to this day, evidence is too limited to support this claim.

This study highlights the complex interplay between an accessory navicular bone and bone stress injury in polycystic ovary syndrome. While accessory navicular bones are a relatively common anatomical variation often detected incidentally, they can contribute to altered biomechanics and, in rare cases, to pain and dysfunction. Secondly, PCOS was considered protective for bone health due to hyperandrogenism and a higher BMI. Now, it is recognised as potentially detrimental due to chronic inflammation, insulin resistance, and vitamin D deficiency.[6] In PCOS, a vitamin D deficit is often encountered and may facilitate its pathophysiology. Hypovitaminosis D is both directly and indirectly linked to numerous aspects (menstrual/ovulatory irregularities, PTH elevation, obesity) of polycystic ovary syndrome, which is, in turn, associated with an increased risk of fractures.[6]

Altogether, these metabolic and hormonal disturbances, as well as the biomechanical abnormalities, could increase the risk of bone stress injuries, though concrete evidence remains limited. Comprehensive and longitudinal research about the impact of PCOS on bone health within the athletic population and the risk of bone stress injuries depending on the type of accessory navicular bone is needed regarding management and injury prevention.

## Conclusion

In conclusion, this case study serves as a reminder for clinicians to look at the bigger picture, including the patient’s profile with overlapping risk factors. Optimise diagnostic and therapeutic management, do not simply assume that an athlete with PCOS has healthy bones, but dig deeper.
